# tRNAs as Antibiotic Targets

**DOI:** 10.3390/ijms16010321

**Published:** 2014-12-25

**Authors:** Shaileja Chopra, John Reader

**Affiliations:** Department of Cell Biology and Physiology, the University of North Carolina at Chapel Hill, 536 Taylor Hall, 109 Mason Farm Road, Chapel Hill, NC 27599-7545, USA; E-Mail: schopra@email.unc.edu

**Keywords:** tRNA, protein translation, antibiotics, stringent response, biogenesis

## Abstract

Transfer RNAs (tRNAs) are central players in the protein translation machinery and as such are prominent targets for a large number of natural and synthetic antibiotics. This review focuses on the role of tRNAs in bacterial antibiosis. We will discuss examples of antibiotics that target multiple stages in tRNA biology from tRNA biogenesis and modification, mature tRNAs, aminoacylation of tRNA as well as prevention of proper tRNA function by small molecules binding to the ribosome. Finally, the role of deacylated tRNAs in the bacterial “stringent response” mechanism that can lead to bacteria displaying antibiotic persistence phenotypes will be discussed.

## 1. Introduction

tRNAs are the key decoding adaptors in the protein synthesis machinery that allows the mRNA genetic code to be translated into a linear sequence of amino acids in a polypeptide chain [1,2]. There are numerous tRNA isoacceptors for each of the canonical 20 amino acids designated by the genetic code [3]. These tRNA species show a significant variety in both base sequence and modifications in both prokaryotes and eukaryotes while still maintaining the ability to bind to the highly conserved ribosome active sites [4]. This essential and ubiquitous nature of tRNAs in protein synthesis make them prime targets for antibiotics with a vast array of natural and synthetic inhibitors that interfere with these components of protein translation. The targeting of these antibiotics is not simply restricted to tRNAs and aminoacyl-tRNAs bound to the ribosome but all the biological processing and tailoring stages that are required to make the aminoacyl-tRNA substrates. tRNAs have also been found to play diverse roles in the cell, such as cell wall biosynthesis, cell membrane permeability and antibiotic persistence, which can also be targeted by antibiotics [5,6]. The purpose of this review then is to describe these tRNA targeting antibiotics and their mechanisms. This may provide more insights into approaches that could be undertaken to design novel anti-infectives for the treatment of diseases.

**Figure 1:**
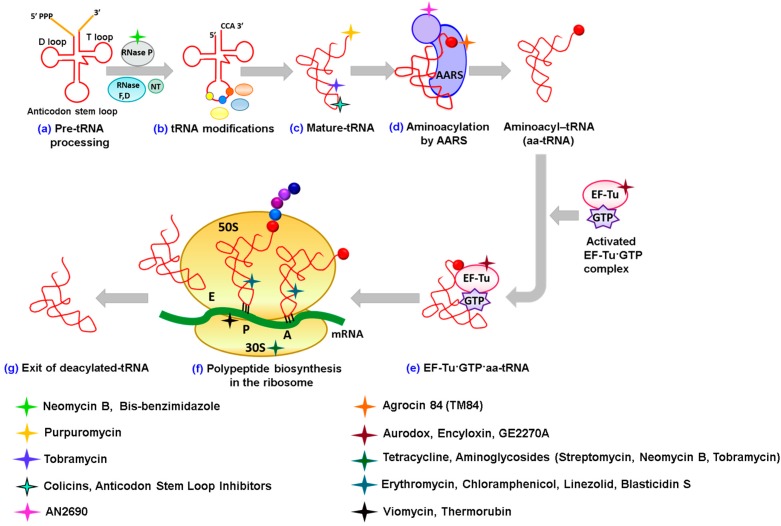
Stages of tRNA biology and protein biosynthesis in bacteria targeted by antibiotics. (**a**) Pre-tRNA transcripts are first processed by ribonucleases (RNase) E or F at the 3'-end, followed by RNase D. Similarly, the 5'-leader sequence is acted upon by RNase P, which is followed by nucleotidyl transferases (NT) for addition of CCA at the 3'-trailer sequence; (**b**) Modification of certain bases takes place using tRNA modification enzymes (mostly in the anticodon stem loop domain) to produce (**c**) the mature tRNA; (**d**) Aminoacyl-tRNA synthetases add amino acids to the cognate tRNA at the CCA 3'-end; (**e**) Activated EF-Tu·GTP complex recognizes the aminoacylated tRNA and transports it to the A-site on the 30S ribosomal subunit; (**f**) Once assembly of the mRNA, fMet-tRNA^Met^ and 50S subunit is complete, the peptidyl transferase reaction starts and initiates the building of the polypeptide chain. Once the ribosome encounters a stop codon, the addition of amino acids to the polypeptide chain halts and the complete polypeptide chain is released; and (**g**) Deacylated tRNA then exits the ribosome at the E site and the ribosome once again dissociates into 30S and 50S subunits. Examples of some antibiotics are shown that inhibit each of these different stages from (**a**) to (**g**).

In order to fulfill their roles in protein synthesis, tRNA transcripts have to be extensively processed by an assortment of enzymes and ribonucleoprotein complexes (Figure 1). The process of tRNA maturation involves five main steps: (a) Removal of the 5' leader sequence from the tRNA; (b) removal of the 3'-trailer sequence by RNase E or F followed by RNase D; (c) Addition of the CCA 3'-end by tRNA nucleotidyl transferases (or repair of this region for those tRNA genes that encode the CCA 3'-end); and (d) Modification of tRNA, such as methylation of tRNA bases, to assist in folding, addition of dihydroxyuridine in the D-loop and addition of pseudouridine in the TψC loop. A majority of modifications take place in the anticodon stem loop as detailed later.

Mature tRNAs then have to be ligated to the correct amino acid catalyzed by the aminoacyl tRNA synthetase (aaRS) family of enzymes [7] (Figure 1). AaRSs all catalyze the tRNA aminoacylation reaction in two steps. Firstly, each canonical amino acid reacts with ATP to form an enzyme bound aminoacyl-adenylate intermediate and inorganic pyrophosphate. Secondly, a correct tRNA isoacceptor bound to the respective aaRS repositions its 3'-end into the catalytic active site and a covalent linkage is initially formed at the 2'-OH or 3'-OH of the terminal adenosine of the tRNA with concomitant formation of AMP [8]. Several mechanisms exist to ensure that the correct amino acid gets attached to the end of the tRNA. For this, tRNA forms extensive interactions with the aaRSs using the acceptor stem, the anticodon loop as well as the D and the TψC arm regions [9]. These interactions allow the enzyme to identify recognition elements of different tRNAs, thereby aminoacylating the correct one. Other mechanisms towards maintaining the fidelity of the genetic code involve hydrolysis of the incorrect amino acids after being interrogated by the proofreading mechanisms of the aaRSs [7,10,11]. This allows for very low error rates to occur during translation.

Once the aminoacylated tRNAs are biosynthesized they rapidly bind to an elongation factor called EF-Tu (elongation factor thermos unstable) and then proceed to the ribosome for protein translation (Figure 1). The bacterial ribosome is a huge 70S ribonucleoprotein complex composed of over 4500 RNA nucleotides and ~50 ribosomal proteins. It consists of two subunits denoted as the 50S subunit (large) that contains the peptidyl transferase center and the 30S unit (small) that binds to mRNA and encompasses the decoding center that monitors the tRNA anticodon-codon interaction. The 70S ribosome has three binding sites for the tRNA molecules, which span the space between the two ribosomal subunits: A (aminoacyl), P (peptidyl) and E (exit) sites. In prokaryotes, translation is started by a 30S subunit bound to mRNA forming an initiation complex with the initiation factors IF1, IF2 and IF3 and fMet-tRNA^Met^ that is positioned correctly at the first codon. There is then a coordinated binding event where 50S subunit joins the 30S subunit with release of the initiation factors leaving the codon bound fMet-tRNA^Met^ at the P-site. Incoming EF-Tu·GTP·aa-tRNA complexes then interrogate the mRNA codon at the A-site of the 30S subunit-decoding center with their anticodon stem-loops (Figure 1). Once the correct pairing of codon to anticodon (often involving tRNA modifications) occurs, the GTP bound to EF-Tu is hydrolyzed and releases the aminoacylated tRNA at the A-site. With the correct tRNAs positioned in the P-site and A-site of the ribosome, the peptidyl transferase reaction takes place so as to add the polypeptide chain from the tRNA at the P-site to the tRNA on the A-site (Figure 1). Then, in a reaction catalyzed by an incoming EF-G·GTPase, the A-site tRNA now ligated to the new polypeptide chain translocates along with the mRNA, making it the new P-site and the deacylated tRNA moves over to the E-site to dissociate from the ribosome [12,13]. The elongation cycle is repeated until a stop codon is encountered leading to the termination stage of translation involving release factors and EF-G, which lead to release of the nascent polypeptide from the ribosome and dissociation of the ribosomal subunits. It is important to note from an antibiotic perspective that although the eukaryotic protein translation machinery works in similar manner to the prokaryotic one, there are significant differences, such as ribosomes size (80S in the case of eukaryotes), differences in ribosomal proteins and translation factors and variation in tRNA number, sequence and modifications [14].

Thus, all these processes where tRNAs are involved can be targets for new drugs. In the sections described below, we aim to summarize the mechanisms of known antibiotics that target tRNAs involved in different processes.

## 2. Antibiotics Affecting tRNA Biogenesis and Modification

### 2.1. Antibiotics Preventing tRNA Maturation

Ribonuclease P is an endoribonuclease that cleaves the 5'-end of pre-tRNA to generate mature tRNA. This is an essential step in the biogenesis of tRNA. A bacterial RNase P holozyme structure has been elucidated from *E. coli* and comprises a catalytic RNA or ribozyme subunit (350 to 450 nucleotides), and a single protein subunit (110 to 150 amino acids) [15]. The RNA subunit is termed M1 and the protein subunit is referred to as C5. The interactions between M1 and C5 are essential for the recognition and binding to its substrates [16]. A comparison of the RNase P subunits between different bacteria indicates only some degree of identity. For example, there is only 30% similarity between the protein subunits of *Bacillus subtilis* and *E. coli.* Importantly, from an antibiotic perspective*,* there exist striking differences between bacterial and human RNase P (which contains a much higher number of protein subunits). These differences between host and microbe make RNase P an attractive candidate for the design of new antimicrobials.

**Figure 2:**
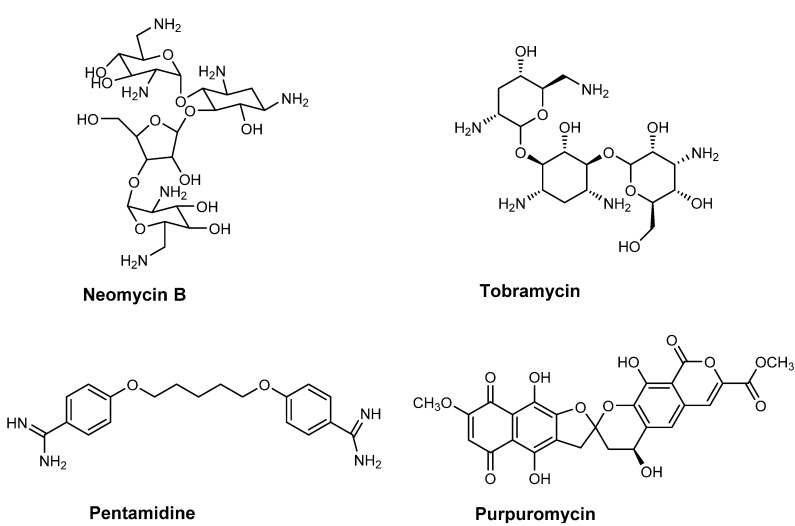
Chemical structures of RNase P inhibitor (neomycin B) and inhibitors that directly interact with tRNA (tobramycin, pentamidine and purpuromycin).

A number of RNase P targeting antibiotics are known, which use a number of different modes of action. Inhibition mechanisms include: Binding to the pre-tRNA substrate and disrupting RNase P substrate recognition; binding to RNase P itself and altering the P RNA conformation or disrupting the association of RNA and protein in the complex; and finally binding and disruption of the enzyme-substrate complex (RNase P·pre-tRNA). Some examples of antibiotics that bind to pre-tRNA substrates include aminoglycosides, which displace functional metal ions, and have been shown to prevent *E. coli* RNase P recognizing the substrate [17,18,19,20]. Specifically, neomycin B binds pre-tRNA with a half maximal inhibitory concentration (IC_50_) of 60 μM) [21] (Figure 2); While derivatives of neomycin with positively charged lysine, arginine or guanidinium groups exhibit IC_50_ values ranging from 0.1 to 6 μM [18,19]. *E. coli* RNase P inhibitors porphines and porphyrins with inhibition constant (*K*_i_) ranging from 1 to 4 μM work in a similar manner displacing metal ions and/or by altering the tertiary structure of tRNA [20,22,23]. Synthetic RNase P inhibitors, such as bis-benzimidazoles have been found to bind to the T-stem of *E. coli* pre-tRNA^Phe^ and prevent RNase P interaction (IC_50_ of 5–20 μM) [24]. Interestingly, peptidyl-transferase inhibitors (e.g., puromycin (IC_50_~3mM); Amicetin and blasticidin S) can also block RNase P activity by binding to pre-tRNA [25,26].

Antisense technology has also been utilized to target the RNA moiety of RNase P itself [27] with inhibitors towards *E. coli* and *B. subtilis* RNase P with IC_50_ values obtained of 2 nM to 1 μM, respectively [27,28]. The P15 loop region of *E. coli* RNase P is important in forming interactions with the 3'-end of the pre-tRNA substrate. Gruegelsiepe* et al.*, synthesized a 14 base oligomer that is stable and resistant to nucleases and demonstrated that it binds irreversibly to RNase P. These* in vitro* studies showed that the antisense oligomer specifically targets the catalytic core (P15 loop) of RNase P and traps it in a partially unfolded state, thereby affecting substrate binding. In addition to this it also affects the coordination of Mg^2+^ ions that are important for catalysis. The success of this technique is due to the accessibility of the oligomer to this P15 loop region. Further investigations of this oligonucleotide* in vivo* conjugated it to an invasive peptide via a monoglycine linker (peptide nucleic acid) to facilitate the uptake of the inhibitor into live *E. coli* cells. Inhibition of growth was observed indicating a successful uptake of the oligomer by the cells and specific targeting of the *E. coli* RNase P. The formation of hybrid-duplexes with RNase P and subsequent partial unfolding of the ribozyme is thought to make the complex more prone to degradation in the cell.

Since bacterial RNase P differs markedly from its eukaryotic counterpart, it is possible to specifically target only the bacterial RNase P. In addition, RNase P is present in relatively lower concentrations as compared to ribosomes [29]. Therefore, a lower concentration of the antisense oligomer would be sufficient to inhibit the RNase P and thereby prevents cell survival. In addition, other potential inhibitor target sites have expanded, such as the identification of the P10/11–J11/12 regions of Type A RNase P [30]. Taken together, direct targeting of bacterial RNase P holds considerable potential.

Recently a potent inhibitor, Ir6Ac (IC_50_ = 820 nM) has also been identified. Ir6Ac is a semisynthetic derivative of irigenol that is produced by plants of the* Leguminosae* family. This compound binds to RNase P with a unique mechanism, which is unlike that of aminoglycosides. This inhibitor not only binds to the RNase P alone (*K*_i_ = 130 nM), but also binds to the RNase P·pre-tRNA^Asp^ complex (*K*_is_ = 480 nM). Contrary to aminoaglycosides, such as neomycin B, Ir6Ac cannot bind pre-tRNA^Asp^ or tRNA^Asp^ alone, but requires the presence of RNase P for its action [31].

### 2.2. Antibiotics Affecting tRNA Modification

tRNAs contain a large number of modified bases that are involved in a variety of roles from structural stability, recognition elements required for discrimination by aaRSs, to hyper modified bases on the anticodon loop of the tRNA allowing a single tRNA isoacceptor to read multiple codons and prevent frameshifting on the mRNA [32]. A number of the tRNA modifications, and their related biosynthetic enzymes, are found in all domains of life while others are conserved amongst bacteria. Despite their ubiquitous nature, very few of these modifications are actually essential for bacteria [33] and so there is a restricted range of candidate biosynthetic enzymes that can be targeted by antibiotics. One noticeable exception is TrmD, a tRNA methyl transferase [34] found to be essential in *Streptococcus pneumoniae* [35], *Bacillus subtilis* [36] and *E. coli* [37]. TrmD methylates the guanine base at position 37 on the anticodon loop to form M^1^G37 on a number of tRNA species, which prevents frameshifting on the mRNA bound at the A-site of the ribosome [38]. This essential enzyme is now under investigation as a promising target for developing antibiotics [39]. Another tRNA modification, which is a potential target for antimicrobials is threonylcarbamoyl adenosine (t^6^A), which is essential and universally conserved in all three domains of life and found on the anticodon loop on tRNAs that decode the ANN codon [40,41]. Recent exciting discoveries have identified the genes responsible for biosynthesizing this hyper modified base opening up the possibility of developing new antimicrobials that inhibit t^6^A’s biosynthesis [42,43]. Although, genes that are essential is an important feature of targeting novel broad spectrum antibiotics, this does not exclude the possibility that some tRNA modifications maybe required to support pathogenic lifestyles of certain bacteria and could be targets for more selective antimicrobials. Indeed, such pathogenic specific antimicrobials maybe beneficial as they would not kill commensal bacterial, which can restrict the growth of newly antibiotic resistant forms of the pathogen taking over the environment. One such example is the 5-methylaminomethyl-2-thiouridine (mnm5s2U34) modification synthesized by the GidA and MnmE enzymes, which form a binary biosynthetic complex [44]. Deletion of these genes that encode these enzymes was reported to attenuate bacterial virulence in a number of important pathogens.

## 3. Antibiotics Having Direct Interactions with tRNAs

### 3.1. Alteration of tRNA Conformation

tRNA structures can be stabilized by metal ions, such as Mg^2+^, and positively charged polyamines, such as spermidine, which bind non-specifically to nucleic acids. Aminoglycoside antibiotics mimic polyamines due to the high number of positive charges and this allows them to bind to the negatively charged backbone of RNA [45,46]. In addition, the flexible structure of these molecules allows them to penetrate the binding pockets and make specific contacts with RNA. One such example is that of tobramycin (Figure 2), which binds to the A-site on the 16S rRNA and thus interferes with the conformational change that occurs at the A-site upon the correct tRNA-mRNA codon interaction [47]. Importantly, tobramycin has also been shown to interact with yeast tRNA^Asp^ and fluorescence anisotropy experiments demonstrated this binding destabilizes the native conformation of the tRNA. Tobramycin binding also leads to a substantial 30-fold decrease in the catalytic affinity (or *K*_m_) of aspartyl-tRNA synthetase (AspRS) for its tRNA^Asp^ substrate resulting in inhibition of the aminoacylation reaction [48]. Detailed studies using single nucleotide resolution SHAPE (selective 2'-hydroxyl acylation analyzed by primer extension) chemistry have further elucidated how specifically tobramycin binding results in the loss of interactions between the T and D loops and eventually the structure of the D-stem [47,49]. Another aminoglycoside Neomycin B (Figure 2) binds to the upper part of the anticodon stem in *E. coli* tRNA^Phe^ at positions G20, A44 and G45 [50]. It obstructs the region of the tRNA involved in the aminoacylation reaction thus inhibiting the phenyl-tRNA synthetase activity.

Another compound, pentamidine (Figure 2) (an aromatic diamine) has been used in the treatment of protozoal infections and binds to tRNAs non-specifically at higher concentrations than those of other antibiotics [51]. Rather than employing electrostatic interactions to bind to tRNA, pentamidine forms hydrophobic interactions and inserts itself into the helical regions of tRNA. This can disrupt the tRNA secondary structure, mask the anticodon loop, and has been shown to lead to the inhibition of aminoacylation in the case of tRNA^Leu^ [52].

### 3.2. Blocking the CCA 3'-End of tRNA

Purpuromycin (Figure 2) is an antibiotic produced by *Actinoplanes ianthinogenes* that has been shown to bind to the 3'-acceptor stem of all tRNAs with high affinity thereby preventing the aminoacylation of tRNA by its cognate amino acid [53,54,55]. The structure of this antibiotic is unique and consists of a naphthazarin and isocoumarin ring systems that are connected to each other by a bis-benzanelated 5,6-spiroketal core (Purpuromycin is active against Gram-positive bacteria, such as *Bacillus subtilis* possessing a minimal inhibitory concentration (MIC) of 0.06 mg/L; *Candida albicans* with MIC of 1 mg/L and protozoa, such as *Trichomonas* sp. A number of synthetic derivatives of purpuromycin have been synthesized to increase activity [56,57,58] and have been proposed to have potential for the topical treatment of vaginal infections [59].

### 3.3. Cleavage of tRNAs

Colicins are antibacterial toxins secreted out into the extracellular medium by members of the enterobacteriaceae family, such as *E. coli* (about 30% of *E. coli* contain them). They are produced as a tool to decrease competition from other bacteria living side by side in the same environment [60]. Several different types of colicins exist and they kill the target cell by various mechanisms, such as DNase activity, RNase activity, depolarization of the cytoplasmic membrane, and inhibition of murein synthesis. Of these, colicins E5 and D are RNases and enzymatically target specific tRNAs and cleave them. This results in the depletion the levels of tRNA required for aminoacylation, consequently impairing protein synthesis and causing cell death.

Colicin E5 RNase targets tRNAs specific for tyrosine, histidine, asparagine and aspartic acid by cleaving anticodon QUN that contains the hyper-modified queuosine nucleotide (Q) at the wobble position 34 [61]. On the other hand, colicin D affects all four isoacceptor tRNAs of arginine at the anticodon loops at positions 38 and 39 [61]. Both the colicin E5 and D exhibit their effect* in vivo* as well as* in vitro*. In addition to the nuclease activity by colicin E5 and D, there exists another type of nuclease called PrrC [61]. These are produced by a clinical strain of *E. coli* in response to a T4 phage infection. PrrC is a suicidal nuclease since it cleaves its own lysine tRNA as a result of the phage infection [62] and it does so at the anticodon loop of tRNA^Lys^ [63]. Thus the modes of substrate recognition by these colicins and their cleavage sites are different. Yet, they achieve the same physiological function of cleaving the tRNAs [62]. This is intriguing since all colicins possess a similar mechanism of entry into the cell. Further investigation in the mode of action of colicins is needed before use as a treatment for infections.

### 3.4. Affecting the Binding of the Anticodon Stem Loop of tRNA

The anticodon stem loop region (ASL) of microbial tRNAs from bacteria and fungi are diverse in RNA sequence as well as containing a range of modifications (Figure 1). Scientists at Trana Discovery Inc., (located at Research Triangle Park, NC, USA, www.tranadiscovery.com) have used modern bioinformatics tools to conduct a detailed analysis of the tRNA of certain organisms with a particular focus on the ASL region of tRNAs. From this analysis, probes that mimic the ALS region have been designed. Using this probe, high throughput sequencing (HTS) analysis has been performed to screen for compounds that can bind with high affinity and specificity to the ASL region of the particular tRNA. Some examples using this novel patented technology include the HTS assay for methicillin resistant *Staphylococcus aureus* that identified compounds that bind specifically to tRNA^Arg^. By utilizing a fluorescein-labeled oligonucleotide mimic of the ASL loop of tRNA^Arg^ on a ribosome scaffold, biochemically active compounds could then be isolated and then tested for dose response [64,65,66]. This general approach is not restricted to bacteria and has been used to identify compounds that bind to the tRNA^Lys3^ of HIV [67,68,69] and also fungal tRNAs from certain plant pathogens. Thus, targeting of the ASL region of tRNAs from specific organisms is an attractive approach for the design of inhibitors of human and animals disease and as well as in agriculture.

## 4. Antibiotics Inhibiting Aminoacylation of tRNA

The aminoacylation of tRNAs by aaRSs has been found to be targeted by a large number of natural occurring antibiotics. This is perhaps not surprising considering the essential role aaRSs play in translation and with typically 18–20 different amino acid specific enzymes per bacterial cell. What is surprising is that very few aaRS inhibitors are actually used in a clinical setting. Pseudomonic acid or mupirocin is one example, yet is one of the most effective topically applied antibiotics used to combat methicillin resistant *S. aureus*. This antibiotic is a naturally occurring isoleucyl-tRNA synthetase inhibitor produced by *Pseudomonas fluorescens* strains and works by docking onto the enzyme catalytic active site and competing with the isoleucine and ATP substrates for binding. This effectively prevents formation of the isoleucyl-adenylate reaction intermediate and therefore aminoacylation of tRNA^Ile^. A number of natural aaRS inhibitors employ similar mechanisms to inhibit their respective aaRSs. Rationally designed stable mimics of aminoacyl-adenylate reaction intermediates have also been studied extensively and prove to be potent inhibitors of their respective aaRS* in vitro*. However, these inhibitors have issues with bioavailability and bacterial uptake and often do not sufficiently discriminate between bacterial and eukaryotic aaRSs for use as an effective antibiotic.

Other aaRS inhibition mechanisms that prevent tRNA aminoacylation include amino acid mimics, non-competitive inhibitors that bind outside of the active site but modulate enzyme activity and antibiotics that directly bind to the substrate tRNA (mentioned earlier in text). We have recently reviewed these aaRS inhibitor mechanisms in detail [70] as have others in a number of excellent reviews [71,72,73,74,75,76]. For the purposes of this tRNA review we will therefore focus on two novel but separate tRNA-dependent inhibition mechanisms employed by antibiotics that target leucyl-tRNA synthetase (LeuRS).

### 4.1. Inhibition by Trapping tRNA in a LeuRS Editing Domain

**Figure 3:**
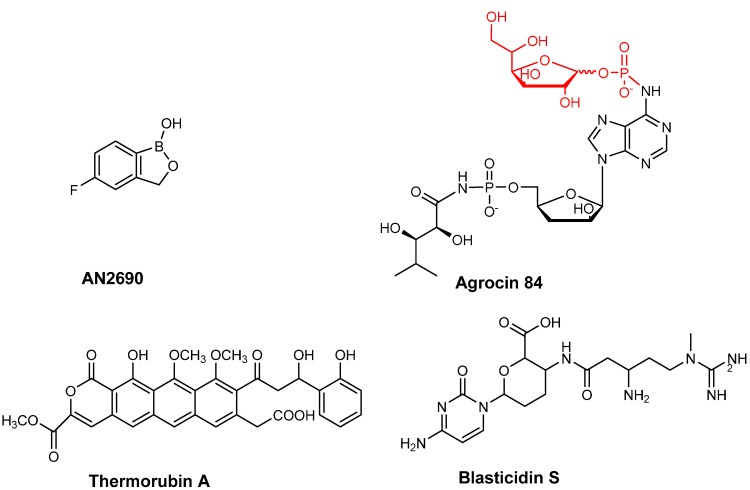
Chemical structures of tRNA based inhibitors of aminoacyl-tRNA synthetases (AN2690 and agrocin 84/TM84) and ribosome (thermorubin and blasticidin S).

A number of aaRSs, which catalyze the aminoacylation of branched chain amino acids, such as LeuRS, valyl-tRNA synthetase (ValRS) and IleRS, possess an additional proofreading domain called the CP1 domain [77,78,79]. This domain is located about ~30 Å away from the aminoacylation active site and is involved in recognizing and hydrolyzing misacylated amino acids on the 3'-end of the tRNA^Leu^. Recently, a pharmaceutical company (Anacor) used combinatorial screening of a library of organoboron compounds and discovered a novel synthetic compound, AN2690 (Figure 3) that inhibited a fungal LeuRS from the yeast *Saccharomyces cerevisiae*. AN2690 (5-fluoro-1,3 dihydro-1-hydroxy-2, 1-benzoxazole) was found to show broad-spectrum activity with minimum inhibitory concentration (MIC) in the range of 0.5 to 1 μg/mL. Importantly, when *S. cerevisiae* colonies resistant to AN2690 were generated, the mutations were all tracked to the *CDC60* gene, which encodes the cytoplasmic LeuRS [80]. Most interestingly the mutations were found to all be located in the LeuRS editing domain [81]. This observation led to the discovery that AN2690 works by binding to the enzyme’s editing domain [80] in a non-competitive manner. Specifically, contacts made with the 2' and 3'-oxygen atoms of the ribose of the 3'-terminal adenosine of tRNA leads to the formation of a stable tRNA^Leu^-AN2690 adduct [80]. This adduct is effectively trapped in the editing domain preventing further rounds of aminoacylation occurring on the enzyme.

Based on this model, further benzoxaborole derivatives have been synthesized that contain the boronic acid core [82]. Substitution of the oxoboroles at the 3, 6 or 7 positions resulted in derivatives possessing MIC values in the range of 2 to 32 μM and IC_50_ values in the range of 5 to 400 nM. AN2690 was found to be most potent of all the compounds and very effective against fungi, yeast, molds or dermatophytes causing skin infections. AN2690 is specifically effective against *Trichophyton rubrum* and *Trichophyton mentagrophytes* the causative agent of onychomychosis, a disease of the human nail bed (an infection that affects approximately 35 million people in the US [83,84]. Moreover, AN2690 has demonstrated superior activity compared to the already available antibiotics, such as ciclopirox, terbinafine and itraconazole. One of the main advantages AN2690 had over these existing antibiotics is that its small size allows it to easily penetrate and reach the nail bed [85]. Further studies on the physiochemical and pharmacokinetic properties of AN2690 have been carried out. After completion of clinical trials, the FDA has recently approved AN2690 (also called tavaborole) [86]. The new drug is marketed as Kerydin, which is a 5% topical solution of oxaborole for the topical treatment of onychomycosis.

The use of this boron family of compounds as an antibiotic is not restricted to fungi, as a derivative of oxaborole has been found to be effective against protozoa [87,88]. These compounds are cyclic boronic esters that are substituted at positions 5, 6 or 7 and are effective against *Trypanosoma brucei* and *Plasmodium falciparum* with IC_50_ values between 0.07 and 9 μg/mL and 120 nM to 5 μM, respectively [88,89,90,91,92,93]. Other compounds with substitution at position 6 by di-substituted aryl or heteroaryl sulfonamides have excitingly been shown to be effective against Gram-positive and Gram-negative bacteria, and also Mycobacterium [82,84,86,94,95,96,97,98].

### 4.2. Inhibition by Trapping tRNA in an “Aminoacylation-Like” Conformation

Agrocin 84 (Figure 3) is a Trojan horse inhibitor that targets the pathogenic strains *Agrobacterium tumefaciens* and *Agrobacterium rhizogenes* that in plants cause crown gall tumors and hairy root condition, respectively. The antibiotic is produced by another agrobacterial strain, *Agrobacterium radiobacter* strain K84 and can act as a natural biocontrol agent and can prevent plant tumors in a number of agriculturally important species [99]. Tumorgenic *Agrobacterial* strains contain a tumor-inducing or Ti-plasmid from which an oncogenic fragment of DNA called T-DNA is injected into wounded-plant cells through a molecular syringe and stably incorporated into the plant genome [100,101]. The expression of the T-DNA genes leads to production of plant hormones as well as carbon-rich compounds called opines, which are secreted. The pathogenic agrobacteria in turn metabolize these giving a rational for the infection process [101,102].

Agrocin 84 acts as a molecular Trojan horse by mimicking a tumor-derived substrate called agrocinopine [103,104] and so hijacks the *A. tumefaciens*’ opine permease to gain access into the interior of the cell [105,106,107]. Once inside, agrocin 84 is processed into a toxic moiety called TM84 that inhibits LeuRS [108,109] (Figure 3). TM84 is structurally similar to the leucyl-adenylate intermediate (Leu-AMP) that is formed in the first step of the LeuRS aminoacylation reaction. One of the key differences is that Leu-AMP possesses an unstable phosphoanhydride bond as opposed to TM84, which contains a stable *N*-acyl phosphoramidate bond [110]. Surprisingly, TM84 is a weak inhibitor of the amino acid activation reaction. This is unexpected as it suggests that TM84 does not behave as a standard aminoacyl adenylate analog discussed earlier. An explanation for this behavior has recently been found that demonstrates that another player is involved in the TM84 inhibition of LeuRS. Biochemical and biophysical analyses have revealed that tRNA^Leu^ is required to form a tight binding complex of *A. tumefaciens* LeuRS·tRNA^Leu^·TM84 (IC_50_ ~1 nM, *K*_iapp_= 0.3 nM) This was also confirmed by the X-ray crystal structure of the complex of *E. coli* LeuRS·tRNA^Leu^·TM84, which demonstrated that TM84 does indeed bind to the catalytic active site; But the CCA 3'-end of tRNA^Leu^ is positioned into the enzyme active site, trapped into an “aminoacylation-like” conformation. Specifically, the terminal adenosine of the CCA 3'-end directly interacts with the TM84 toxin. Additionally, TM84 forms interactions with the highly conserved, mobile KMSKS loop of LeuRS, thereby allowing the loop to adopt a closed-conformation encapsulating TM84 and explaining the high affinity of the toxin in the presence of tRNA^Leu^ [109]. The two novel tRNA-dependent inhibition mechanisms employed by TM84 and AN2690 highlighted here may well provide promising stepping-stones to development of tRNA-dependent inhibitors of the other amino acid specific aaRSs.

## 5. Antibiotics Affecting Elongation Factor EF-Tu

After aminoacylation of each tRNA by its respective bacterial aaRS, all the aminoacyl-tRNA products bind to a single, highly conserved, 44-kDa protein called EF-Tu (elongation factor thermo unstable). EF-Tu is a GTPase that only binds to the aminoacyl-tRNAs in a GTP bound form and then transfers the aminoacyl-tRNAs to the A-site of the ribosome and is therefore critical to the protein translation machinery. Upon codon-anticodon recognition in the ribosome, EF-Tu undergoes a conformational change leading to the hydrolysis of the active EF-Tu·GTP complex to the inactive EF-Tu·GDP complex [111,112,113]. This results in the release of EF-Tu from the ribosome, which is then available for regeneration with GTP [114]. Inhibitors of EF-Tu are divided into two broad categories. Of these, kirromycin and enacyloxin affect the EF-Tu·GTP or ·GDP complex [115,116], while pulvomycin and GE2270 (also called as MDL 62,879) prevent the formation of a stable complex of aminoacyl-tRNA with EF-Tu·GDP/GTP [117,118,119].

Kirromycin is an antibiotic that binds to the ribosome·aa-tRNA·EF-Tu·GDP complex. This results in inhibition of the release of the EF-Tu·GDP complex from the ribosome. Failure of the release of EF-Tu from the ribosome does not affect the binding of aminoacyl-tRNA to the A-site of the ribosome, but blocks the subsequent peptide bond formation step [115,116]. Thus, kirromycin is a potent inhibitor of the EF-Tu dependent protein translation reaction. Aurodox (also called mocimycin) is a *N*-methyl derivative of kirromycin and is produced by *Streptomyces goldiniensis* [120], and crystal structure of *Thermus thermophilus* EF-Tu with GDP and aurodox in a 1:1:1 ratio at 2 Å resolution indicates that binding of aurodox resembles the EF-Tu·GTP bound conformation [121]. Additionally this stabilization of the EF-Tu·GTP complex occurs in the absence and presence of aminoacyl-tRNA. Thus kirromycin strongly affects the conformation of EF-Tu by interfering with its allosteric function and consequently protein translation. Enacyloxin IIa is produced by *Frateuria* sp*.* W-315 and is active against both Gram-positive and Gram-negative organisms [122,123,124]. This antibiotic affects the interaction between EF-Tu and GTP by retarding the dissociation of GTP from the complex. This results in alteration of the conformation of aa-tRNA, thereby leading to the deacylation of the aa-tRNA that is bound to the EF-Tu·GTP complex. This consequently blocks polypeptide chain formation. Enacyloxin IIa also weakly affects the binding of aa-tRNA to the A-site on the ribosome [125,126].

GE2270A is a thiazolyl peptide antibiotic that is active against Gram-positive bacteria. Crystal structure of *E. coli* EF-Tu·GDP·GE2270 complex has confirmed that this compound directly competes with aminoacyl-tRNA for the same binding site on EF-Tu. It also blocks the GTP to GDP conformational change in EF-Tu. A comparison of binding modes indicates that GE2270 therefore has a different binding mode compared to kirromycin and enacyloxin IIa but is limited as an antibiotic due to its low solubility [127,128,129]. Pulvomycin has a similar mechanism as GE2270 wherein it prevents that binding of aa-tRNA to EF-Tu [117,118,119]. Additionally, it prevents the binding of amino acids to the 3'-end of the tRNA. Overall, these inhibitors block protein synthesis by either directly or indirectly affecting the binding of aa-tRNA to EF-Tu and consequently its function in the ribosomal machinery.

## 6. Targeting tRNAs in the Ribosome

There exist three tRNA binding sites on the ribosome: an A-site for the incoming aminoacylated tRNAs, a P-site for the peptidyl-tRNAs and an E site where the deacylated tRNAs exit. The tRNA species bound to these sites adopt a large number of different conformations during the three-stage translation process of initiation, elongation, and termination. As a consequence of this highly complex and essential process, there exists a vast array of natural and synthetic drugs that target different stages of the process binding to the ribosome. Many of these toxins directly interact or interfere with tRNA species in mechanisms ranging from prevention of tRNA anticodon loop binding to mRNA in the decoding center to prevention of aminoacyl-tRNA movement into the P-site the peptidyl transferase center (PTC). The space available in the article limits the detail in which we discuss the large number of antibiotics targeting the ribosome but we direct the reader to a number of excellent reviews recently written on the topic [130,131,132,133].

Initiation of protein synthesis involves, the formation the 70S subunit of the ribosome along with the initiator fMet-tRNA and start codon of mRNA positioned at the P-site. Drugs that inhibit this step are broadly classified either as 50S or 30S inhibitors. Examples of antibiotics binding to the 50S subunit of the ribosome include macrolide (e.g., erythromycin) [134], lincosamide (e.g., clindamycin) [135,136], streptogramin (e.g., dalfopristin), amphenicol (e.g., chloramphenicol) and oxazolidine (e.g., linezolid) classes of antibiotics [137]. These include inhibitors that prevent the binding of the initiator tRNA at the P-site (e.g., oxazolidines) [138,139,140] and antibiotics that prevent peptide bond formation and/or the translocation of tRNA from the A-site to the P-site on the ribosome (e.g., macrolide, lincosamide and streptogramin class of antibiotics) [141,142]. This eventually leads to interference with the elongation step and thus the inhibition of protein translation.

Similarly, antibiotics inhibiting the 30S subunit include tetracyclines and aminocyclitols (made up of aminoglycosides and spectinomycin). Tetracyclines bind to the 30S subunit and block the binding of aminoacylated tRNAs to the A-site of the ribosome [143,144]. Spectinomycins bind the elongation factor and thereby prevent the binding of peptidyl-tRNAs [145,146]. Aminoglycosides (e.g., streptomycin, gentamycin and kanamycin) bind to the 16S rRNA component of the 30S subunit. Binding results in alteration of the interaction between the mRNA codon and the ribosome, thereby affecting the binding interaction of the incoming aa-tRNAs [147,148]. This results in generation of mistranslated proteins that are toxic to the cell. Since aminoglycosides intake can result in production of toxic proteins, this can affect cell wall and cell membrane compositions, thereby increasing the membrane permeability and subsequently increasing uptake of the drug in the cell [149,150] (although uptake is limited under anaerobic conditions). Hence aminoglycosides are bactericidal, while most of the protein translation inhibitors are bacteriostatic. Aminoglycosides also include the 2-deoxystreptamine class of antibiotics (e.g., neomycin, paramomycin, dibekacin and amicakin). These drugs are potent inhibitors of the translocation reaction and they also prevent subunit separation during ribosome recycling [151]. Additionally, these they cause errors in translation by stabilizing the binding of non-cognate tRNAs to the mRNA [137,147].

Other examples of antibiotics binding to the 30S subunit include tuberactinomycins, thermorubins (Figure 3) and blasticidin S (Figure 3). Tuberactinomycins (e.g., viomycin and capreomycin) are natural antibiotics that bind the interface of the 30S and 50S subunit of the ribosome and thereby inhibit translocation. These drugs have been used for the treatment of *Mycobacterium tuberculosis* infections including the multi-drug resistant types [152,153,154]. Thermorubins also bind at the interspace spanning the two ribosomal subunits and inhibits the binding of fMet-tRNA to the P-site [155].

Blasticidin S is an antibiotic produced by *Streptomyces griseochromogenes* [156]. BlaS has been found to be a potent inhibitor of both prokaryotic and eukaryotic cells. MIC value of BlaS towards *E. coli* cells was 50 μg/mL and towards mammalian cells was 2 to 10 μg/mL. Unlike other blasticidins, BlaS possesses a unique mechanism of inhibition of the protein translation machinery. Analysis of the crystal structure of BlaS bound the 70S ribosome complex revealed that BlaS binds to the 50S subunit of the ribosome at the P-site and not at the A-site like other Bla antibiotics [157,158]. Upon binding the P-site, BlaS bends the CCA 3'-end of the tRNA bound at the P-site to the A-site resulting in greater than 7 Å shift in the ribose phosphate backbone of the base C75 of the tRNA. This results in a decrease in the flexible movement of the CCA 3'-end of the tRNA, an important feature required by translation. In addition, BlaS also interferes with ligands binding to the A-site [159].

## 7. Antibiotics Affecting Non-Canonical Roles of tRNA

### 7.1. Inhibition of Amidotransferases

**Scheme 1:**
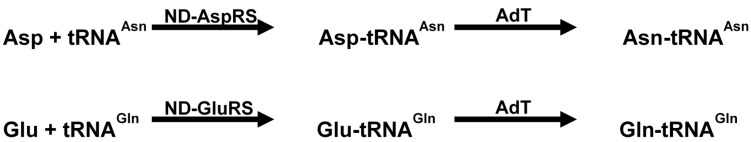
Reaction catalyzed by non-discriminating AspRS/GluRS and amidotransferases (modified from Tambula-Hansen* et al.*, [161]).

Genomic studies have shown that certain microorganisms lack glutaminyl-tRNA synthetase (GlnRS) and asparaginyl-tRNA synthetase (AsnRS) [160]. These organisms rely on an alternative pathway for production of Gln-tRNA^Gln^ and Asn-tRNA^Asn^. The pathway first involves production of Glu-tRNA^Gln^ and/or Asp-tRNA^Asn^ by a non-discriminating synthetase (ND) followed by transamidation to Gln-tRNA^Gln^ and Asn-tRNA^Asn^ by glutamine amidotransferase [161]. This enzyme is a heterotrimeric protein encoded by the genes *gat*C, *gat*A and *gatB* (Scheme 1).

Recently synthetic inhibitors of the amidotransferase (adT) enzyme have been synthesized. These inhibitors, aspartycin and glutamycin mimic the 3'-end of the mis-aminoacylated tRNA. Both aspartycin and glutamycin are competitive inhibitors of *Helicobacter pylori* (*H. pylori*) GatCAB with respect to Asp-tRNA^Asn^ [162,163]. Glutamycin also resembles a derivative of puromycin (naturally produced by *Streptomyces alboniger*). A number of puromycin derivatives have been synthesized to test their effect on *H. pylori* GatCAB complex [164]. Therefore, aspartycin and glutamycin and their derivatives could serve as promising future antibiotics of those pathogenic bacteria that encode the GatCAB complex.

### 7.2. Affecting Cell Wall Biosynthesis and Cell Permeability

Peptidoglycan is the major component of the Gram-positive bacterial cell wall and is composed of alternating *N*-acetyl glucosamine (GlcNAc) and *N*-acetylmuramic acid (MurNAc) connected by a β-1, 4 linkage. The GlcNAc and MurNAc subunits are linked to short peptides of variable sizes [165]. In some pathogenic bacteria, such as *Streptococcus pneumoniae* and *S. aureus*, these peptide chains contain additional Ala-Ala and Ser-Ala inter-subunit crosslinks [166]. Interestingly, two types of enzymes, FemXAB and MurMN ligases are involved in the formation of these additional cross-linked subunits and both of them utilize aminoacyl-tRNAs for the generation of these amino acid crosslinks [167]. FemXAB is involved in adding (Gly) 5 cross-linked subunits in methicillin resistant *S. aureus* using Gly-tRNA^Gly^ [167]. MurMN belongs to the same family as FemXAB ligases and is involved in adding Ala-Ala and Ser-Ala in *S. pneumoniae* using Ala-tRNA^Ala^ and Ser-tRNA^Ser^, respectively. Thus, these enzymes and their aminoacyl-tRNA substrates are important targets for antibiotic design against multi-drug resistant bacteria [168]. Attempts have been made for the rational design of FemX inhibitors by employing modelling and mutagenesis studies of tRNA^Ala^. Using this approach, an analogue of aminoacyl-tRNA linked to 1, 2, 4 oxazolidine ring has been synthesized that inhibits Fem transferases in *Weissella* *viridescens* (FemX_Wv_ (IC_50_ = 1.4 μM) [169]. Similarly, MurM from *S. pneumoniae* was inhibited by a 2'-deoxyadenosine 3'-phosphonate analogue [170].

## 8. tRNAs and Antibiotic Persistence and Resistance

### 8.1. Deacylated tRNA and the Stringent Response

Nutritional stress or inhibition of the aminoacylation reaction of aaRSs results in the accumulation of deacylated tRNA. These uncharged tRNAs then bind to the A-site on the ribosome and are recognized by RelA and/or SpoT, which then trigger the stringent response mechanism [171,172,173,174,175,176]. The resultant effect of the stringent response mechanism is elevated levels of alarmones, such as guanosine tetraphosphates (ppGpp) and guanosine pentaphosphates (pppGpp), which then inhibit RNA polymerase [171]. Thus inhibition of protein synthesis subsequently leads to down regulation of RNA synthesis [177]. Recently a novel compound relacin has been found that inhibits RelA and the subsequent production of (p)ppGpp [178]. The resultant effect of the stringent response leads to a series of physiological changes in the bacterium that aids in survival under adverse conditions, such as exposure to antibiotics. Several examples of persistence of microorganisms under stress conditions have been observed [179]. One of the effects involves formation of biofilms, which involves self-assembly into highly organized, surface attached and matrix encapsulated structures. Both, *Mycobacterium tuberculosis* and* Mycobacterium smegmatis* form biofilms under conditions of stress and can tolerate about 50 times the MIC of antituberculosis drugs, such as isoniazid and rifampicin [180,181,182,183], thus making them less sensitive to these antibiotics. In some bacteria, such as the human pathogen *Pseudomonas aeruginosa*, quorum sensing gets elevated upon expression of the *relA* gene [184]. Elevated levels of (p)ppGpp also results in increased virulence, pathogenesis and survival in the host. This has been demonstrated in *Pseudomonas* sp., *Samonella* sp., *Vibrio cholera* and *Legionella* sp. [185,186,187,188]. Increase in the production of secondary metabolites and antibiotics has also been reported in some organisms, such as *Streptomyces coelicolor* [189]. Some other persistence responses include initiation of sporulation in *Myxococcus* sp. due to increased levels of (p)ppGpp [190,191] and formation of nodules in soil bacteria, such as Rhizobia, in response to nitrogen limitation [192,193]. Targeting deacylated tRNA bound to the ribosome and/or interfering with the deacylated tRNA-dependent RelA activity is promising target to prevent the antibiotic persistence phenotype seen in a number of important human pathogens [178].

### 8.2. Bacterial Resistance Mechanisms

Resistance to antibiotics that interfere with tRNA biology can be mediated by a variety of different mechanisms. Some of these involve alterations in membrane permeability to impair the influx of the drug or to have an active efflux of the drug from the cell. Examples of these include chloramphenicol, erythromycin, neomycin, streptomycin and spectinomycin [132,194,195]. Another highly prevalent mode of resistance is mutation of the target sites and occurs in most examples of antibiotics that affect tRNA function (as covered in this review); examples of which are: Blasticidin S, chloramphenicol, erythromycin, linezoloid, neomycin, spectinomycin, streptomycin and viomycin [196,197,198,199]. Another example is that of structural modifications in EF-Tu (due to mutations in the* tuf B* gene), which leads to kirromycin resistance. Of course, spontaneous resistance mutations can appear in aaRSs genes in organisms exposed to aaRS targeting antibiotics, which we recently reviewed elsewhere [70]. Of the tRNA-dependent aaRS inhibitors mentioned here, AN2690 resistant *C. albicans* strains appeared as a result of a single point mutation in the LeuRS CP1 editing domain [81]. While agrocin 84 resistant agrobacteria has been reported in the field through transfer of the pAgK84 plasmid, encoding the TM84 self-immunity LeuRS AgnB2 [108], from the biocontrol organism *Agrobacterium radiobacter* strain K84 to the plant pathogen *A. tumefaciens* [200].

Modification of the binding site of the antibiotic is another mechanism to decrease the affinity of the drug. For example, methylation of rRNA by rRNA methyltransferases confer resistance to aminoglycosides, chloramphenicols, oxazolidinones and some macrolides [201,202]. This consequently obstructs tRNAs from their function in the ribosome.

In order to lower the effective concentration of the drug, some microorganisms increase the expression of target or a mimic of the target so as to lower the effective concentration of the drug and thus leave some target molecules uninhibited. For example, overexpression of the rRNA fragment that resembles h34 of the 16S rRNA is involved in conferring resistance to spectinomycin [203]. Similarly, increased production of EF-Tu has been observed in response to the EF-Tu inhibitor amythiamycin [204]. Alternatively, proteins are produced that bind to the target site and protect it by decreasing the affinity of the drug. For example, Tet O and Tet M proteins are produced in response to tetracycline inhibition, which bind to the tetracycline-stalled ribosomes and sterically dislodge the antibiotic from the binding site [205,206]. Another mode of antibiotic resistance is by degrading or modifying the antibiotic itself. Chemical modifications, such as phosphorylation, acetylation, adenylation, glycosylation, and hydroxylation of antibiotics, have been reported for chloramphenicols, aminoglycosides, macrolides and tetracycline. This results in a reduced affinity of the drugs to their ribosomal targets [207,208,209,210,211,212].

## 9. Conclusions and Future Directions

The central role of the numerous tRNA species in the microbial protein synthesis apparatus makes these ribosome associated adaptor molecules prominent targets for antibiotics. In this review, we have highlighted that antibiotics can also target a significant number of key processing and tailoring steps that are also essential for tRNAs to carry out their role in translation. With the ever-looming specter of microbial antibiotic resistance threatening the usefulness of standard medical treatments against human pathogens, there is a growing need for the development of new anti-infectives. This search has to confront a decreasing number of new essential targets to explore and also find antibiotics less prone to imparting resistance in the microbes on which they are trained. In this respect, the novel tRNA-targeting antibiotics discussed in this review hold out some hope. From the design of inhibitors targeting ASL region of tRNA [213] and essential tRNA modification enzymes, to the discovery of new tRNA-dependent inhibition mechanisms against aaRSs, such as those employed by AN2690 [80] and agrocin 84 [109], the future for developing tRNA targeting antibiotics looks promising. Finally, tRNAs have been found to play an increasing number of non-canonical roles in microbes and these maybe another rich vein to mine novel drugs and targets [169,170].
